# Shaping understandings through reflexive practice: Learnings from participatory research on aging with multiple sclerosis

**DOI:** 10.1186/s40900-024-00614-x

**Published:** 2024-07-31

**Authors:** Sofie Olsgaard Bergien, Lasse Skovgaard, Josephine Lyngh Steenberg, Maria Kristiansen

**Affiliations:** 1 The Danish Multiple Sclerosis Society, Poul Bundgaards Vej 1, Valby, 2500 Denmark; 2 Department of Public Health & Center for Healthy Aging, University of Copenhagen, Fredericiagade 18, Copenhagen K, 1310 Denmark

**Keywords:** Aging, Multiple sclerosis, Participatory research, Reflexivity, Patient association, Partnerships, Older adults

## Abstract

**Background:**

Participatory research has gained traction as an approach to unlock perspectives when creating scientific knowledge and to facilitate societal changes. By conducting research *with* people, participatory research strives to engage individuals’ perspectives in designing, conducting, and disseminating the research. Nevertheless, few studies have unpacked how understandings of the studied phenomenon are shaped among diverse research partners and, concurrently, how different perspectives are combined. Nested within an overall participatory mixed methods study on aging with multiple sclerosis (MS), this qualitative study explores how understandings of aging with MS are shaped in encounters between university researchers, older adults with MS, and employees in a patient association.

**Methods:**

The study was collaboratively conducted in Denmark by three research partners: a group of older adults with MS, employees in a patient association, and university researchers. Data on how different understandings of aging with MS were represented and shaped during the three-year research process was generated through field notes, meeting minutes, focus group interviews, and individual interviews. The collected data was analyzed through a thematic network analysis.

**Results:**

The study demonstrates how different understandings of aging with MS were represented among the research partners when the research was initiated. These understandings were shaped prior to —and, therefore, outside—the research setting, drawing from the research participants’ lived experiences, professional backgrounds, and organizational cultures or situated in larger societal narratives. Through a process centered on reflexivity among the engaged research partners, the understandings of what it means to age with MS was shaped and re-shaped and eventually merged into a more dynamic understanding of later life with MS where different perspectives could co-exist.

**Conclusion:**

The findings demonstrate that research partners, including older adults with MS and employees from a patient association, brought diverse understandings to the study. Reflexive practices enabled these perspectives to co-exist, enhancing engagement and transparency, and fostering a dynamic understanding of later life with MS. This highlights the value of reflexivity in evolving complex understandings within participatory research.

**Supplementary Information:**

The online version contains supplementary material available at 10.1186/s40900-024-00614-x.

## Abstract (plain language summary)

In recent years, participatory research has been increasingly utilized in various research fields (e.g., aging research) with the ambition of engaging a diverse group of research partners to leverage their perspectives and contributions. This approach aims to form a more nuanced understanding of the studied phenomenon and to identify insights useful for practice However, working in diverse research teams have also been found to be complex, and it is poorly understood how the perceptions of the different research partners shape the final research product. Based on a study conducted in collaboration with employees in a patient association, older adults with multiple sclerosis (MS), and university researchers, the present study aims to unfold how perspectives of aging with MS are represented and shaped doing a participatory research period. It occurred over a three-year period where data was collected through field notes, meeting minutes, focus group interviews, and individual interviews. The findings highlight how research partners represent different understandings of the studied phenomenon, which is embedded in their social, cultural, and professional background and which potentially influence their expectations of and contributions to the research process. Furthermore, the study demonstrates how engaging in a critical dialogue about expectations and understanding of the studied phenomenon can provide insight into which perceptions are represented, making the participatory research process more transparent. Lastly, the study conveys how such critical dialogue facilitates understandings and perspectives evolving during the research process into a potentially more dynamic understanding of the studied phenomenon.

**Supplementary Information:**

The online version contains supplementary material available at 10.1186/s40900-024-00614-x.

## Background

In recent decades, participatory research has been increasingly used in various research fields with the ambition of engaging a diverse group of research partners (e.g., community members, patients, and private or public organizations) to leverage their perspectives, form a more nuanced understanding of the studied phenomenon, and identify insights useful for practice [1, 2]. According to Andrea Cornwall and Rachel Jewkes, participatory research can be understood and applied as a research methodology, not referring to a particular theory, method, or toolset but rather a broader concept defined in terms of: “…who defines research problems, and who generates, analyses, represents, owns and acts on the information which is sought.” [2] (p. 1668). Following this definition, participatory research can be viewed as a flexible and iterative process [3, 4]. Consequently, the engagement of research partners in a particular research process cannot be predetermined, as the context and characteristics of the partnership influence how collaboration unfolds [4, 5]. Instead, researchers working in a participatory research setting should take into account the specific context they are working in and strive to empower those who are normally the “objects’ of research to become ‘agents’ with the ability to analyze their own situation and co-design future initiatives [2].

Within aging research, scholars have argued that applying participatory approaches may be particularly important because it can challenge widely held assumptions about later life [4, 6, 7]. Where research within the field of aging historically has focused on the “vulnerability” of aging, participatory research holds the potential to challenge this perspective [6, 7]. By engaging older adults as active research partners and enabling their contribution with their own understanding of later life, participatory research can provide a more in-depth and nuanced understanding of what it means to age—including both the agency and strengths of a population [4, 6, 8–10].

Working in participatory research setting often means that lived experiences should work together with professional insights or expertise – coming from e.g. researchers or medical or social care professionals [11–13]. While this combination enriches both the research and the process of implementing findings in care settings or institutional practices [6, 14, 15], conducting research within a diverse partnership consisting of university researchers, citizens, and public or private institutions (e.g., patient associations) also entails potential challenges [9, 10, 16]. Studies in the field of participatory aging research, for instance, stress that a diverse partnership means that many understandings and positions are brought into the research setting, potentially creating a complex environment that is difficult to monitor and understand [10, 17, 18], with the risks of imbalanced power relations affecting which perspectives dominate the final research outcome and of neglecting the lived experiences over professional or academic insights or preferences [3, 18–20].

While the overall reasons for conducting participatory research are to let different perspectives and understandings shape the study [10, 21], the social dimensions of how research partners’ perspectives are shaped by the context they are coming from or their encounters within the collaboration are often neglected [19, 22]. Today, few studies unfold how research findings are shaped by the research partners engaged in the research process, as well as how their different perspectives are combined during the research process to prevent one perspective from overshadowing another. To contribute to this field, this study aims to determine how understandings of the studied phenomenon are shaped among research partners engaging in a participatory research setting by drawing on data collected during the mixed-method participatory research study, “Aging with MS” [23, 24].

### Context of the paper

**Fig. 1:**
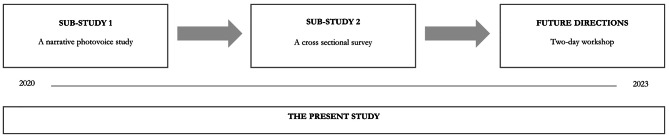
“Aging with MS” study design

The research presented in this paper builds on data generated during the participatory mixed methods “Aging with MS” study conducted between October 2020 and October 2023 in Denmark. The aim of the overall study was to contribute to the field of research concerning later life with multiple sclerosis (MS) by exploring how aging with MS unfolds in people’s everyday lives. As the scientific literature on aging with MS, especially in the context of people’s everyday lives, is limited the “Aging with MS” study was conducted based on a participatory methodology [2], aiming to involve patients and the public as research partners to define the focus of the research. Further this methodology were applied in order to not only inform new scientific knowledge but also provoke societal innovations by empowering the engaged research partners to seek for solutions or future initiatives target later life with MS [2, 13]. The “Aging with MS” study were designed as an exploratory sequential mixed methods design [25], with a qualitative study (sub-study 1) followed by a quantitative study (sub-study 2) (Fig. 1). For a more detailed description of sub-study 1 and 2 please see the following references [23, 24]. In order to support that the findings from sub-studies 1 and 2 were combined into joint recommendations for future research and support offerings targeting older adults with MS, a two-day workshop was held for all engaged research partners. During this workshop findings were discussed and specific recommendations for how the patient associations in the future should focus on later life with MS were crafted. Concurrently with the two sub-studies and the final workshop, the study presented within the present paper were conducted, aiming to unfold the participatory process and to understand how different perceptions of the later life with MS were shaped among the research partners and formed into what ended up being the final research outcome (Fig. 1).

#### The engaged research partners

Three research partners were engaged in the “Aging with MS” study: university researchers, employees in a patient association, and a group of older adults with MS (aged 65 years or older). The older adults living with MS were represented by a nine-member advisory board. Advisory board members were recruited through the patient association’s social media, newsletters, social workers, and psychologists, as well as its magazine. One hundred and twenty older adults with MS registered to be part of the advisory board. Nine people were purposefully sampled [26] to ensure diversity in terms of gender, age, residence, educational level, and physical function. All selected members had MS and were aged between 65 and 78 years; four members were male and five were female. One advisory board member had previous experience with participatory research and eight participants did not. During the three-year research period, four members withdrew from the advisory board. Of these, two members withdrew due to MS-related symptoms (primary cognitive challenges), one withdrew due to not having their expectations fulfilled, and one left due to an illness in their family. Among the four dropouts, two were replaced with new members with matching background characteristics. The two remaining members were not replaced as they withdrew close to the end of the research period.

The patient association employees were engaged in the study through the participation of 55 employees with professional backgrounds in psychology, social work, communication, event planning, research, administration, and marketing. Employees were continuously recruited via email or face-to-face invitations during the research period. The employees were recruited on an ongoing basis when their expertise was needed. None of the employees invited to participate in the research refused, and employees only withdrew from the study if they ended their employment at the patient association. Lastly, the first author of the study was affiliated with both the university and the patient association, thus bridging the practical and academic worlds.

#### How the research partners worked together

Throughout the study period, the three research partners worked in collaboration to design, conduct, analyze, and disseminate the results from sub-studies 1 and 2. This collaboration were embedded in a participatory research methodology and by guided recommendations from the field, including how to foster trustworthy relationships, use inclusive language, ensure participants do not feel disempowered, and encourage a flexible approach through shared dialogue and reflexivity [10, 15, 16, 27]. As participatory research within the present study, are defined and applied as a flexible iterative process, that should by shaped by its context and determined in collaboration with all engaged research partners, the activities and approaches used for engagement and collaboration differed depending on the preferences of the participating research partners. For instance, a two-day workshop including accommodation was arranged when the advisory group engaged in data analysis in sub-study 1, while the online platform Miro was used to create the desired flexible work environment for the employees. Finally, phone calls, email correspondence, and personal visits were used to involve those participants who could not participate in the more formal activities due to personal or professional reasons. For instance, when an advisory board member fell ill during the research period, phone calls, a home visit, and email correspondence kept that member engaged. In total, one workshop and five meetings with the patient association employees and one workshop and three meetings with the advisory board prior to the joined dissemination workshop.

In the first two stages of the study (Fig. 1), separate meetings were held between the first author and the advisory board and the first author and the employees. This arrangement was made under the assumption that the patient association employees were in a position of power that could overshadow the older adults’ perspectives and consequently skew the research toward a professional viewpoint [1, 28]. However, both groups were presented with each other’s input and given the opportunity to comment and discuss ideas and suggestions. In the third phase of the research process, all three research partners participated in a joint workshop to discuss the findings of sub-studies 1 and 2 (Fig. 1).

Finally, to ensure that the advisory board members and employees could share their thoughts and critically reflect upon their own and others’ perspectives on an ongoing basis, the first author strived to facilitate shared and individual reflection sessions inspired by reflexive practices [29–31]. Reflexivity can be defined as a process which invites people to critically questioning their “… own way of thinking, assumptions and underlying patterns of values and world views” [32] (p. 420), which can potentially yield insights into how pre-existing beliefs and contexts affect data generation and interpretation [33, 34]. In the realm of qualitative research, reflexivity is often deemed essential for researchers as it allows them to examine how their assumptions and values might have impacted the creation and interpretation of research data [34, 35]. Furthermore, in participatory research, reflexivity has been highlighted as an approach that may facilitate the sharing of different perspectives represented in a research setting, giving the involved research partners the opportunity to learn from each other [32]. Consequently, the present study aimed to facilitate critical dialogue in which research partners’ assumptions, values, prejudices, and attitudes were not only questioned by the researchers, but also by themselves. The engaged research partners were invited on several occasions to share their thoughts about later life with MS, working in a participatory research setting, and their expectations for the study. These reflections either took the form of shared group discussions or were individual exercises where the research partners wrote their reflections and shared them in the group afterward if they wished. In every instance, data was collected from the exercises to gain insights into the research partners’ thoughts about the research process as well as their contributions to the study.

To report on the engagement of research partners in the participatory study ‘Aging with MS’, we employed the Guidance for Reporting Involvement of Patients and the Public (GRIPP2, short form) (see Additional file 1).

## Methods

### Data generation

To collect data on how an understanding of later life with MS was shaped within the participatory mixed methods study ‘Aging with MS,’ multiple methods (e.g., field notes, meeting minutes, focus groups, and individual interviews) were applied across the entire research process (see Fig. 1). Combining field notes, meeting minutes, focus groups, and individual interviews allowed us to triangulate methods, generating data from different perspectives and gaining insights into the context, the research partners’ understandings of aging with MS, and how these understandings were formed during the research process [36].

From fall 2020 to fall 2023, field notes were collected during individual visits or conversations at the office, phone conversations, email correspondence, workshops, and meetings with the advisory board or employees. Furthermore, formal meeting minutes were taken when meetings or workshops were facilitated, and written contributions by the research partners were likewise collected.

In addition, two focus group discussions were conducted among advisory board members a year into the study period. The focus groups were moderated by JLS, who had not previously been attached to the study. This decision was made to create a ‘safe space’ where members of the advisory board felt they could freely discuss their thoughts while remaining anonymous to the first author. The group discussion was structured using a guide constructed in collaboration between all authors that was inspired by existing literature, with the primary aim of understanding how advisory board members experienced engagement as research partners and how the dynamic between them unfolded [37]. The group discussions were audiotaped, transcribed, and anonymized before the first authors received them. Shortly after the two focus group discussions, a semi-structured individual interview with the first author was facilitated by JLS. The interview aimed to gain insight into the first author’s considerations about conducting participatory research, as well as how she perceived the collaboration between research partners unfolding. Instead of solely gathering the first author’s thoughts about the research process through field notes and reflections, the semi-structured interview allowed her thoughts to be questioned and further elaborated upon. The interview was structured by an interview guide inspired by existing literature on potential dilemmas, barriers, and methodological and ethical reflections relevant to researchers working with participatory research [10, 12, 29]. The interview was audiotaped and verbally transcribed.

During the research period the collected data were at an ongoing basis read through and informing the following focus in terms of question to ask or specific situations to be aware of during the partnership.

### Data analysis

The data analysis was initiated by typing field notes and transcribing audio recordings using NVivo 12. This process was performed parallel to the data generation, as preliminary findings were important for the continuous reflection on how perspectives and understandings of later life with MS were shaped over time. After the data generation, all data was reread to gain an understanding of the empirical data collected and for the first author to become familiar with the material. An analysis was then performed, guided by the steps of an thematic network analysis [38]. First, data was reduced into smaller meaningful units by applying a coding framework focusing on the three research partners and their understandings of later life with MS. Second, themes were extracted from the coded text segments and organized into coherent groupings [38]. To understand if and how the perceptions of each research partner evolved over the three-year research period, particular attention was given to the timing of data collection and which research partner it related to. After all themes were identified, they were placed in relation to how they emerged across a timeline and grouped into organizing themes, namely the research partners’ understanding of later life with MS and the circumstances shaping their perceptions. In order to cross-validate and enrich findings, data generated at approximately the same point in the research process were compared to look for differences or similarities in, for instance, the meeting minutes, field notes, or interview data. During the analysis, all authors of the paper discussed the emerging themes to enhance reflexivity [39]. The findings will be presented by drawing on empirical examples from the data material, through the following headings: [1] older adults’ representations of aging with MS, [2] employees’ representations of aging with MS, and [3] mediating diverse understandings of aging with MS as a university researcher.

## Results

The analysis unfolded how the research partners engaged in the participatory study “Aging with MS” represented varied perspectives of what later life with MS may entail. These different perspectives – situated in existing social narratives of aging, lived experiences, organizational cultures, and professional backgrounds or expertise – were particularly noticeable in how the research partners perceived later life with MS, often characterized within one of the two dichotomies “vulnerability” or “agency”. Where the older adults with MS quickly found a way where stories about both vulnerability and agency could co-exist in their representation of later life with MS, many of the patient association employees stuck more strongly to their perception of later life being predominantly described in terms of vulnerability and decline. Thus, while the understandings of later life with MS varied among the research partners and at times seemed contradictory, the analysis outlines how the practice of reflexivity enabled them to evolve their perceptions as the research progressed. This allowed for a more dynamic understanding where different perspectives and understandings could co-exist.

### Knowledge about later life with MS situated in lived experiences and professional insights

During the initial conversations between the first author and members of the advisory board, most of the members expressed a two-fold understanding of what it means to age with MS. On the one hand, many participants considered aging with MS to entail a life of opportunities and agency despite their possible physical or cognitive challenges. The representation of what later life with MS entails, was typically embedded within the members’ individual lived experiences but often also referred to as an “atypical” way of aging with MS. Referring to themselves as atypical, the older adults often emphasized their physical state (e.g., “(…) not that disabled” [Field Notes, Fall 2021]), or they considered themselves to be better at managing challenges compared to other older adults with MS. This perception of later life situated in personal lived experience was also reflected in how they perceived their role within the research setting as well as their perception on what should be the main aim of the study. As one advisory board member explained to the first author, he did not only want to focus on the terrible parts of his life “…but that he wanted to tell the story of the people who are having good experiences” (Field Notes, Spring 2021). Furthermore, this representation of themselves as being “atypical,” in opposition to a more typical older adult with MS, unveiled a general understanding of old age with MS as being characterized by physical decline, cognitive challenges, and dependency on others. When asked to reflect on the typical older adult with MS, the majority of the members within the advisory board referred to “decreased mobility,” facing a “lack of network,” “not being social,” or “needing help” (Field Notes, Fall 2021). These descriptions were in contrast to the members’ views of their own lives with MS, which, for instance, one participant described as “us sitting here and not being that heavily affected by our MS” (Field Notes, Fall 2021).

Having faced this apparent dichotomy in the description of later life with MS between “oneself” and “others” on an ongoing basis, the first author was prompted to ask the advisory board why they thought that these two opposing views on aging with MS were prevalent. One participant reflected that one’s “positive” view on their own circumstances could partly be a “coping mechanism—to underestimate your handicap” to help manage the difficult circumstances of aging with MS by telling themself that despite an everyday life with challenging situations, others were doing worse than them. Another participant suggested that healthcare professionals were projecting such perspective and that their neurologist often informed them that they were not among the individuals who were the most affected by MS.

The doctors tell you, that you are not one of the worst affected—because you do not have assistive devices. It is the doctors who decide whether you are considered heavily affected by the disease or not, as they will tell you so. The system tells you whether you are lucky or unlucky. You become defined by your appearance. And we believe what the doctor says (Field Notes, Fall 2021).

This dichotomy in understanding later life with MS – being either heavily affected by the disease or not - was likewise present among the patient associations employees, although the employees’ perceptions were predominantly focused on describing later life with MS in terms of “vulnerability” and “decline”. When asked to articulate their understanding of aging with MS in a workshop at the beginning of the research process, the employees’ descriptions of later life with MS were dominated by statements such as “extremely challenged cognitively,” “isolated,” “heavily affected by their MS,” and “dependent on others” (Field Notes Workshop, Spring 2021). Although some employees across professions described the group as being “very engaged and active” and “reflective and reconciled about their situation,” these perceptions were not the prevailing view of later life with MS among the employees. Furthermore, some employees phrased aging with MS in opposition to “normal aging.” As one employee elaborated,

The disease has made its marks on the body and the mind. [It is] a picture, which shows marks of inefficiency and inactivity. Compared to people of 65 + years of age without sclerosis, who play golf and run around (…) people with MS being 65 + years of age do not have the same opportunities, as the disease has consumed the body (Meeting Minutes, Spring 2021).

This understanding of later life with MS as being in opposition to “normal” aging was found to be important for the employees’ expectations for what kind of knowledge the research should produce as well as how new insight should be applied. For example, when preliminary results from sub-study 1 were presented, some employees emphasized that “they would like more focus on how older adults with MS are different from ‘healthy peers,’” as “it [the research findings] sounds a lot like ‘normal’ aging” (Field Notes, Spring 2022). Furthermore, some employees emphasized that for the research findings to be valuable for them and their work, they required insights into those who “need[ed] their help the most.” As articulated by one employee working in social or psychological counseling for people with MS, “… the picture must not be too rosy” when the research results were disseminated, as they had experienced a “less rosy story” when working with aging individuals with MS (Field Notes, Spring, 2022). Another employee working within marketing and fundraising similar explained that a substantial part of their work was to communicate “the severity of MS, as this is an important message for the [name of patient association]—as people prefer to give money to a severe disease” (Field Notes, November 2021).

Although the employees’ understanding of aging with MS reflected their individual roles within the association and the prevalent mandate of their profession and culture, it was common across professions, that the employees positioned themselves as those who should provide support for people living with MS, creating a need for clearly defined problems that they could address, which reflected their expectations of what the study research should achieve, namely specific knowledge on the difficulties of later life with MS.

### Forming a more dynamic understanding of later life with MS

As the research progressed and the research partners were engaged in working with, discussing and interpreting research data, it became apparent that especially the members of the advisory board started to form a more dynamic understanding of later life with MS. This was particularly clear at a workshop with the aim of discussing and interpretating qualitative data generated in sub-study 1 (Fig. 1). Grounded within common reflections on older adults with MS and their reflections on the empirical data, the advisory board’s perspectives on aging with MS started to evolve to represent a more dynamic description of later life with MS, where both vulnerability and agency could co-exist and where the story about “us” and the “others” became less prevalent. One participant summarized the essence of the workshop: *“*As you age you gain experiences (…) but there is also a sorrow in getting older, you no longer can run, hike on Kilimanjaro or play with your grandkids. (…) Aging with MS is a paradox” (Meeting Minutes, Fall 2021). One woman who subsequently evaluated the workshop, had positioned herself as “atypical” and “not that disabled” a month earlier, yet she concluded that working with interview data about other older adults’ experiences had made her realize that her situation was not as unique as she had thought:

Our eyes are opened to this [aging with MS] not just being one dimensional but having, really, a lot of dimensions. But it is also confirmed that one fits into one of these narratives [interview data from sub-study one]. At least I did (Focus Group Discussion, Fall 2021).

As the research process progressed, advisory board members often represented this new understanding of later life with MS as being a paradox or having manifolded stories. For instance, it was important for the advisory board that the dissemination of the research findings reflected the complexity and the many nuances of later life with MS. As one woman concluded, “It is important that the narrative on aging with MS does not become overly negative, such that it becomes excessively sad to reflect oneself in. But it cannot become overly happy either” (Meeting minutes April 2022). Although the participants developed a somewhat shared understanding that aging with MS is a paradox, they still conveyed different understandings of the nuances of later life with MS that reflected their individual situations. Some participants believed that aging with MS was dominated by loneliness, others highlighted their cognitive challenges and other comorbidities, and yet others focused on the importance of staying invested in society or planning their future. While each understanding of aging with MS was individual and unique, they embraced a more dynamic perception, where both agency and vulnerabilities could co-exist in later life with MS.

In opposition to the members of the advisory board, the majority of employees from the patient association held a more consistent understanding of aging with MS. Where the members of the advisory board highlighted that stories on agency and the opportunities could co-exist in describing later life with MS, the employees saw stories about agency in later life with MS to mainly have a role in providing younger people living with MS with “…hope – that life becomes more than MS” (Meeting Minutes, Spring 2022), and kept requested more insight into the ‘vulnerable’ sides of later life to better support the specific group of older adults living with MS. employees’ their professional backgrounds, their roles within the organization, and expectations for how they should apply the final research results at times, became a barrier to accepting and engaging with research findings or inputs unaligned with their understandings of aging with MS. For example, an employee questioned the first author regarding data on well-being “… as [they] had heard from the psychologists that they [the members] had struggled greatly” (Field Notes, May 2021). Or as a fundraising employee explained the first author, “vulnerable” groups were more attentive if the patient association were to receive money from outside grant holders (Field Notes, November 2021).

However, although some of the employees were asking for insight about the vulnerable sides of later life with MS or the differences between “normal” aging and aging with MS, the reflexivity that emerged during workshops or meetings also sparked reflections among other employees regarding whether their workflow, professional insights, and day-to-day contact with specific groups of members could hinder them from developing a more nuanced understanding of what aging with MS may entail. As noted during a workshop centered on the employees’ perception of aging with MS, “(…) a participant says that they probably do not involve [people with MS] as much as they should, because they [the employees] have become ‘too’ professionally knowledgeable” (Meeting Minutes, Spring, 2021). At the same workshop, another participant wondered if the work within the organization might only give them insights into a small group of people living with MS:

In the best of worlds, we want to be an association relevant for everyone with [multiple] sclerosis [being more than 18,000 in Denmark], but perhaps we are only that for 4,000 people [those using the patient association and the offerings available for members]. Quite a few members do not use us and, thus, we do not learn much about them (Meeting Minutes, Spring 2021).

Despite some employees reflected that their professional background and focus as a patient association might hindered them in getting insight about some people living with MS, it was still difficult to others when research data were contradicting their understanding of later life. This especially became clear when the research findings from sub study 1 were to inform the aim of sub study 2. Where the findings of sub study 1 represented the many nuances of later life with MS – being both agency and vulnerability, these findings were not accepted by all patient association employees because they did not align with their professional experiences. While the first author decided “to trust her research findings” and the older adults’ representations of later life with MS, she acknowledged that it was necessary to address the employees’ expectations in the research process to better maintain their active collaboration.

The questionnaire [planned for sub-study 2] should map who is susceptible to having challenges [in participating in the selected activities], as this would imply that the [patient association’s name] can fulfill its role as a patient association by designing support offerings to those in need (…). It is, however, not certain that this is faithful to the participants’ narratives [from sub-study 1], which to a large extent center on how they have learned to handle and navigate everyday life with MS (…). On the other hand, there are factors that challenge this group. And despite the survey exploring in depth these challenges, it does not imply that this should dominate the overarching story (Field Notes, Spring 2022).

Hence, to accommodate the employees’ request for more insights on the “vulnerable” aspects of aging with MS, sub-study 2 was – in collaboration with both the advisory board and the employees - designed to provide insights on areas in which the group required the support of the patient association, making the findings directly useful for potential service providers. Furthermore, to address the employees’ request for knowledge that resembled “normal” aging less, the first author consulted advisory board members to determine how their representation of later life and the findings of sub-study 1 could be combined with the employees’ request. One advisory board member advised the first author to accept that aging with MS is similar to normal aging and “that growing older is like for peers, but having MS just adds an extra dimension to it” (Field Notes, Summer 2022). Based on this dialogue with the advisory board, the first author acknowledges the employees desire to use “normal aging” as a point of references. Upon receiving comments from employees related to the findings and results overly resembling “normal” aging, the first author for instance highlighted that older adults with MS may find meaning and joy in the same things as their peers. However, these older adults may require support due to their MS to engage more fully in a life they find meaningful. After the final workshop in the third stage of the research process (Fig. 1), it became evident that the employees had, to some degree, embraced a perception of later life encompassing both agency and vulnerability. This were for instance became apparent when a group of employees approached the first author with the idea of dedicating a members’ magazine to aging with MS centered around cases from the research and through their stories “…unfold the many nuances which exist in later life with MS” (Fall, 2023). The magazine would thus not only provide hope to younger people living with MS, but also allow older members to see their own circumstances reflected, providing them with inspiration for daily activities and management strategies.

## Discussion

In participatory research involving both private and public research partners, it is inherent—or even a methodological premise—that the research process is influenced by the partners’ understandings of the study phenomenon situated in their lived experiences or professional insights [1, 28]. This study found that involving older adults with MS and patient association employees led to different, and sometimes conflicting, perspectives on later life with MS, often framed as either vulnerability or agency. Such differences have earlier been described as a potential barrier as some perspectives risk overshadowing others [18, 40–42]. Previous research has for instance described that service providers’ understandings of what constitutes useful knowledge can be a barrier to the representation of lived experiences and contributions by non-professional research partners [12, 17, 18]. As individuals in a participatory setting, tend to align their actions and contributions with those in power [43], disputes within research partnership can potential lead to hindering people with lived experience from redefining their situation, thus perpetuating narratives of later life already dominated by stories of decline, dependency, and vulnerability [6, 7]. Similar findings were also present in the present study, in terms of how some employees’ professional backgrounds and organizational contexts shaped a particular understanding of later life with MS, entailing vulnerability and specific needs, which then made it difficult for them to adapt to and implement findings that did not align with their prior perceptions.

To navigate differences between research partners and prevent some perspectives from suppressing others, several suggestions and calls for action have been made [18, 40–42, 44]. For instance, researchers have stressed the need for shared dialogue, where research partners can equally share perspectives to shape a joint understanding [18, 40–42]. Conversely, researchers have argued that if consensus and agreement are too easily achieved, it may indicate that the partnership has lacked room for truly sharing different perspectives and contributions that may entail contradictions, tensions and developments over time [44]. Instead of aiming to create a frictionless atmosphere where agreement and consensus are seen as the main success criteria or end points, researchers should make room for dissensus and disagreements among research partners [43, 44]. In this study, reflexivity was found to facilitate the co-existence of disagreement and differences among the research partners, including university researchers, older adults, and employees. This practice allowed them to gain insights into and critically examine the origins and implications of their varied understandings of later life with MS. Over time, reflexivity helped the research partners develop a more dynamic understanding of later life with MS, where both stories of agency and vulnerability could co-exist. These findings align with Christopher Kelty’s description of how perplexity and dissensus within a participatory research setting can indicate a truly collaborative process, where diverse perspectives and understandings of the world contribute to a new and more nuanced comprehension of the study’s phenomenon [44]. While it is crucial to acknowledge that, in order to create an environment where research partners can embrace each other’s differences, they must also feel that their own perspectives are recognized and respected. In some instances, power imbalances may cause individuals with lived experiences to perceive peaceful and respectful dissent as insufficient, leading them to advocate for more radical, possibly confrontational forms of dissent to effect change. While our study demonstrates how the practice of reflexivity can support mutual curiosity and space for differing perspectives, it is equally important to recognize that there can be situations where engaged research partners may not desire peaceful co-existence of perspectives, as they feel it may overshadow their unique viewpoints and contributions. Therefore, while promoting reflexivity and the co-existence of differences, it is essential to remain open to stronger or less peaceful forms of dissent when necessary to ensure all voices are genuinely heard and valued.

In addition to supporting the co-existence of differences between the patient association’s employees and the older adults with MS, reflexivity also facilitated the co-existence of different perspectives and nuances within the two groups. For instance, aligned with existing research [19, 28, 45], the present study demonstrates how the older adults with MS represented different understandings of later life with MS. These findings highlight, as also stressed in the existing literature, the importance of researchers being aware that individuals engaged in research projects, despite sharing characteristics like age, illness, or geographical location, do not necessarily hold a homogeneous understanding or perception of the studied phenomenon. Therefore, it is crucial to engage a diverse sample of older adults [19, 46, 47]. However, the findings of this study further emphasize the importance of ensuring that potential differences within a diverse sample can co-exist during collaboration without some perspectives potentially overshadowing others. While existing studies in participatory research have argued that reflexivity can help researchers navigate the complexity of working with partners from different backgrounds [12, 40, 41, 48, 49], this study demonstrates how other research partners—including older adults—can also benefit from reflexive practices by learning about each other’s differences and finding ways for their diverse perspectives to co-exist.

### Methodological considerations

The data presented within the present study was generated through a triangulation between qualitative methods, which potentially increased the trustworthiness of the findings by allowing the phenomenon to be studied from different angles [50]. Furthermore, a strength of the study is that the data was generated across a three-year period, allowing the study to unfold how understandings are shaped and evolved over time. However, the study also entails potential limitations. As mentioned, four advisory board members withdrew due to MS-related symptoms or family illness. This suggests that older adults with severe MS or caregiving responsibilities for sick family members may have been underrepresented on the advisory board. On the other hand, it may also be perceived as unavoidable that some participants withdrew during the lengthy three-year research period and the older adults who did not withdrew from the advisory group did represent a broad group of older adults with MS (e.g. some with comorbidities, some with a severely ill spouse, some living alone, some with a low income, some with cognitive difficulties, and some with advanced MS). Furthermore, it may be argued that the partnership between the patient association employees, the older adults with MS, and the researcher was challenged by the employees and the advisory board not meeting until the third stage of the research process (Fig. 1), which prevented these two research partners from communication during the conduction of sub-studies 1 and 2. However, due to the differences in power between the engaged research partners, we do believe it has been a strength to the study as it has a created a space where both the older adults and the employees could contribute with their insights and understanding without overshadowing one another. Moreover, this design allowed the study to unfold how complex and nuanced understandings of a phenomenon are not only shaped through ongoing communication, but also by separated practices of reflexivity Hence, this design means that the academic researchers hold a great responsibility in facilitating the partnership ensuring that understandings and contributions are merged. Although this study addresses a partnership between three research partners, only the academic research team contributed to the analysis and writing of this paper due to a limited time schedule and lack of financial resources to engage the employees and the older adults in the writing process. However, three of the four authors are affiliated with the patient association, and the advisory board was presented with the final analysis and offered the opportunity to comment and suggest adjustments to the paragraphs. None of the nine board members accepted this invitation, with the argument that the level of the English language was not accessible for them. The study’s timeline and our financial resources did not allow us to translate the text into Danish or use other co-creating tools to engage the advisory board in drafting the manuscript. As this factor created a power imbalance in the dissemination of the present paper, we encourage future studies to engage all research partners in terms of data generation and when interpretation of the findings. Lastly, although the present study was conducted in a Danish context, the findings may be relevant to researchers working in partnership with older adults and their potential service providers in other settings. However, it is essential to acknowledge that different settings and cultures involve unique circumstances that always must be considered.

## Conclusion

The findings of the present study illustrate how each research partner, including older adults with MS and employees from a patient association, brought different understandings of later life with MS to the research, influenced by their professional roles and lived experiences with MS. These perspectives were often represented by perceiving later life with MS in terms of either agency or vulnerability. Although the research partners did not form a joint perspective of what it means to grow older with MS, they created a common research contribution that represented their different interpretations and perspectives. Through reflexive practices, their diverse perspectives on later life with MS were able to co-exist, allowing the research partners’ differences to form a more dynamic understanding of later life with MS. Based on these findings, the study highlights that reflexivity may support a complex and dynamic understanding of the studied phenomenon, where differences can co-exist within a participatory research partnership.

### Electronic supplementary material

Below is the link to the electronic supplementary material.

Supplementary Material 1

## Data Availability

The datasets generated and/or analyzed during the current study are not publicly available to avoid compromising the anonymity of the people engaged as research participants. However, the data is available from the corresponding author by request.

## Abbreviations

MS: Multiple sclerosis
