# Supplementary material for: Shaping understandings through reflexive practice: Learnings from participatory research on aging with multiple sclerosis
Source: Res Involv Engagem. 2024 Jul 31;10:78. doi: 10.1186/s40900-024-00614-x (PMC11293046; doi:10.1186/s40900-024-00614-x)
Supplement: Supplementary file 1 — Supplementary Material 1 [file 40900_2024_614_MOESM1_ESM.docx]

| **Section and topic** | **Item** | **Reported on page No** |
| --- | --- | --- |
| 1: Aim | Report the aim of PPI in the study | 6,7 |
| 2: Methods | Provide a clear description of the methods used for PPI in the study | 7-10 |
| 3: Study results | Outcomes—Report the results of PPI in the study, including both positive and negative outcomes | 2,4,13-20 |
| 4: Discussion and conclusions | Outcomes—Comment on the extent to which PPI influenced the study overall. Describe positive and negative effects | 2-4, 20-24 |
| 5: Reflections/critical perspective | Comment critically on the study, reflecting on the things that went well and those that did not, so others can learn from this experience | 23-24 |
| Staniszewska S, Brett J, Simera I, Seers K, Mockford C, Goodlad S, et al. GRIPP2 reporting checklists: tools to improve reporting of patient and public involvement in research. Res Involv Engagem. 2017 Dec;3(1):13. | | |

**Additional File 1: GRIPP2-SF**
